# Ensemble coding of multiple facial expressions is not affected by attentional load

**DOI:** 10.1186/s40359-024-01598-9

**Published:** 2024-02-27

**Authors:** Yujuan Liu, Luyan Ji

**Affiliations:** 1 Department of Psychology and Center for Brain and Cognitive Sciences, School of Education, Guangzhou University, 510006 Guangzhou, China; 2 Center for Cognitive and Brain Sciences, Institute of Collaborative Innovation, University of Macau, Macao, China

**Keywords:** Ensemble coding, Attentional load, Facial expressions, Emotion perception, Dual-task, Mean emotion, Individual emotion

## Abstract

Human observers can extract the mean emotion from multiple faces rapidly and precisely. However, whether attention is required in the ensemble coding of facial expressions remains debated. In this study, we examined the effect of attentional load on mean emotion processing with the dual-task paradigm. Individual emotion processing was also investigated as the control task. In the experiment, the letter string and a set of four happy or angry faces of various emotional intensities were shown. Participants had to complete the string task first, judging either the string color (low attention load) or the presence of the target letter (high attention load). Then a cue appeared indicating whether the secondary task was to evaluate the mean emotion of the faces or the emotion of the cued single face, and participants made their judgments on the visual analog scale. The results showed that compared with the color task, the letter task had a longer response time and lower accuracy, which verified the valid manipulation of the attention loads. More importantly, there was no significant difference in averaging performance between the low and high attention loads. By contrast, the individual face processing was impaired under the high attention load relative to the low attentional load. In addition, the advantage of extracting mean emotion over individual emotion was larger under the high attentional load. These results support the power of averaging and provide new evidence that a rather small amount of attention is needed in the ensemble coding of multiple facial expressions.

## Introduction

The world is filled with a vast amount of information that contains many similar items, such as a flock of birds, a clump of trees, and a group of people. We can combine these items and extract the summary statistical information (e.g., mean) from them, which is called ensemble coding [1, 2]. Previous studies have revealed that the mean information can be discriminated, reported, and reproduced from multiple levels of stimuli, including lower-level features, such as orientation [3], spatial position [4], and the size of circles [5, 6], as well as higher-level objects such as facial expressions [7, 8]. Ensemble coding of facial expressions has been proven to be rather precise and flexible. Observers can extract the mean emotion (i.e., the average intensity of individual expressions) from a large number of emotional faces presented for a short amount of time (e.g., 16 faces in 500 ms [8]), while they retained little information about the individual faces in the set [8, 9]. However, it is still unclear and debated how we extract summary statistics from multiple facial expressions. One remaining question is about the role of attention in perceiving mean emotion from multiple facial expressions.

The findings that observers can extract mean emotion when limited viewing time is available [9], when multiple faces are crowded in the periphery [10], or when averaging was not explicitly required [8, 11] provide indirect evidence for relative independence between ensemble perception and attention. Some studies, which more directly manipulated attention, have shown that when attention is directed away, ensemble coding of low-level features can still be established [4, 12, 13]. For instance, even though attention was mostly allocated to the targets during the multiple-object-tracking task, the performance of identifying the mean location (centroid) of a few missing distractors at the end of each trial was well above chance [4]. Likewise, the ensemble structure of multiple Gabor patches in the background could be accurately perceived in the demanding tracking task [12]. The mean emotion of a set of faces could also be extracted when attention was maintained at a low level [14], emphasizing again a possible dissociation between this extraordinary visual ability and attention. Specifically, the performance of discriminating mean emotion from four target faces in the periphery was above chance and was comparable at an attended and unattended location, using the spatial cueing paradigm [14].

On the other hand, some studies argued for the dependence on attention in ensemble coding of multiple levels of stimuli [15–17]. For example, Jackson-Nielsen and colleagues [17] showed that participants were inattentionally blind to summary statistics of color or size of letters when attention was focused on recalling the letters. In addition, the performance of letter recall was worse in the dual-task condition where the color diversity or mean size of letters had to be reported concurrently relative to the single-task condition (letter recall only). Taken together, it was suggested that ensemble coding of color and size consumed some attentional resources [17; cf., 13]. A similar cost of dual-task was found in the perceptual averaging of faces [15]. When the single task asked participants to evaluate the emotion of the post-cued face after seeing a pair of two faces within the same visual hemifield, the ratings were impacted by the other face, suggesting an implicit averaging of faces [18]. However, when both the emotion and orientation of faces had to be perceived in the dual-task, implicit emotion averaging did not occur anymore, which implied dependence on attention in ensemble coding [15].

One reason for the somewhat mixed results reported above might be that different levels of attentional loads were used in diverting attention away from the features or objects to be averaged. Similar debates occur in the processing of a single face [19, 20]. Some studies showed similar brain responses to the unattended and attended emotional faces [21, 22]; while some found that unattended emotional expressions induced weaker or even eliminated responses in the brain relative to the attended ones [23–25]. Based on the load theory of attention [26, 27], when emotional expressions are task-irrelevant, if attention is not sufficiently exhausted for the target task (i.e., low load), spare attentional resources would spill over to process the task-irrelevant (and also called unattended) emotional expressions, and thus emotional effect could still be observed. By contrast, if the attentional load is high, few attentional resources would be available for the irrelevant processing, and the emotional effect would be largely diminished and even disappear. Similarly, when emotional expressions are task-relevant and are required to be extracted, an additional perceptual task that consumes different levels of attentional resources might also result in different impacts on the emotional processing of faces.

In the current study, we directly manipulated the attentional load in a dual-task and aimed to examine whether and how ensemble coding of multiple facial expressions would be impacted by the different levels of attentional load. Here, we used the term attentional load rather than perceptual load [26, 27], considering the physical stimuli we presented were the same but the processing demands were different [28–30]. Specifically, participants completed the mean emotion task and a concurrent color (low attentional load) or letter task (high attentional load) [31, 32]. Both sets of happy faces and angry faces with various emotional intensities were used, similarly as in [34–36], to explore the potential differences in the ensemble coding of positive versus negative facial expressions. We also asked participants to do a single task where only the mean emotion judgment was required (no attentional load). It is to be noted that the low and high attentional load used here was relative and did not necessarily occupy the full range of attentional loads. We hypothesized that if ensemble perception of multiple facial expressions was not modulated by attentional load, it would suggest that emotion averaging requires a rather small amount of attention if any. If ensemble perception was systematically modulated by attentional load, that is to say, performance was the worst under high attentional load, and the best under no attentional load, it would suggest that emotion averaging requires quite a large amount of attention. Whether there was any difference between averaging positive and negative faces, and whether it would be modulated by attentional load was exploratory in the current study, and we did not have specific hypotheses since it has not been directly tested and the limited related studies have not been conclusive so far [33–37].

Apart from the mean emotion task, we additionally had an individual emotion task as control, to examine whether ensemble perception of multiple facial expressions would be differently impacted by attentional load (if any) compared to emotional processing of individual faces. Considering the previously demonstrated “power of averaging”, namely ensemble coding can be more precise than individual coding due to the cancellation of random errors in individual measurements when averaged [1, 14, 38], we hypothesized that attentional load would have a smaller effect on mean emotion processing than individual emotion processing.

## Method

### Participant

Twenty-five healthy students from Guangzhou University participated in this study, and one had to be excluded due to low accuracy (at chance level). The sample size of 24 was determined prior, targeting the interaction effect between attentional load (high, low) and task type (mean, individual) in the ANOVA for the performance of emotion judgments. The power analysis was based on the effect size reported in a previous study with a similar design [14] (examining the interaction between levels of attention and type of tasks, ${\eta }_{p}^{2}$ = 0.16), with *α* = 0.05, power (1-*β*) = 0.95 and the number of measurements = 4, using G*Power 3.1.9.7. The correlation among repeated measures (0.5) and the epsilon for sphericity correction (1) was set to be the default. This analysis estimated a sample size of 13 participants, and we decided to enlarge our sample size to ensure sufficient randomization of block orders. All participants (19–25 years, age: *M* = 21 years, *SD* = 1.8; 16 females) were right-handed, had normal or corrected-to-normal vision, and reported no problem with color blindness or color weakness. After obtaining informed written consent, participants completed the experiment and were paid 30 RMB or one credit per hour. The research was carried out under the Declaration of Helsinki and was approved by the ethics committee of Guangzhou University. All participants were naive to the purpose of the experiment.

### Stimuli

Four young faces, two males (Y46M, Y55M) and two females (Y14F, Y17F) were selected from the Tsinghua Facial Expression Database [39]. Each face identity displayed angry, neutral, and happy (smiling with a closed mouth) expressions. The hair, ears, neck, and other external information were cropped, and face contours were preserved. All images were converted to greyscale and scaled to the same level of brightness and contrast. Each face image was displayed on a homogenous black background with a viewing angle of 3.13°×3.13°.

**Fig. 1:**
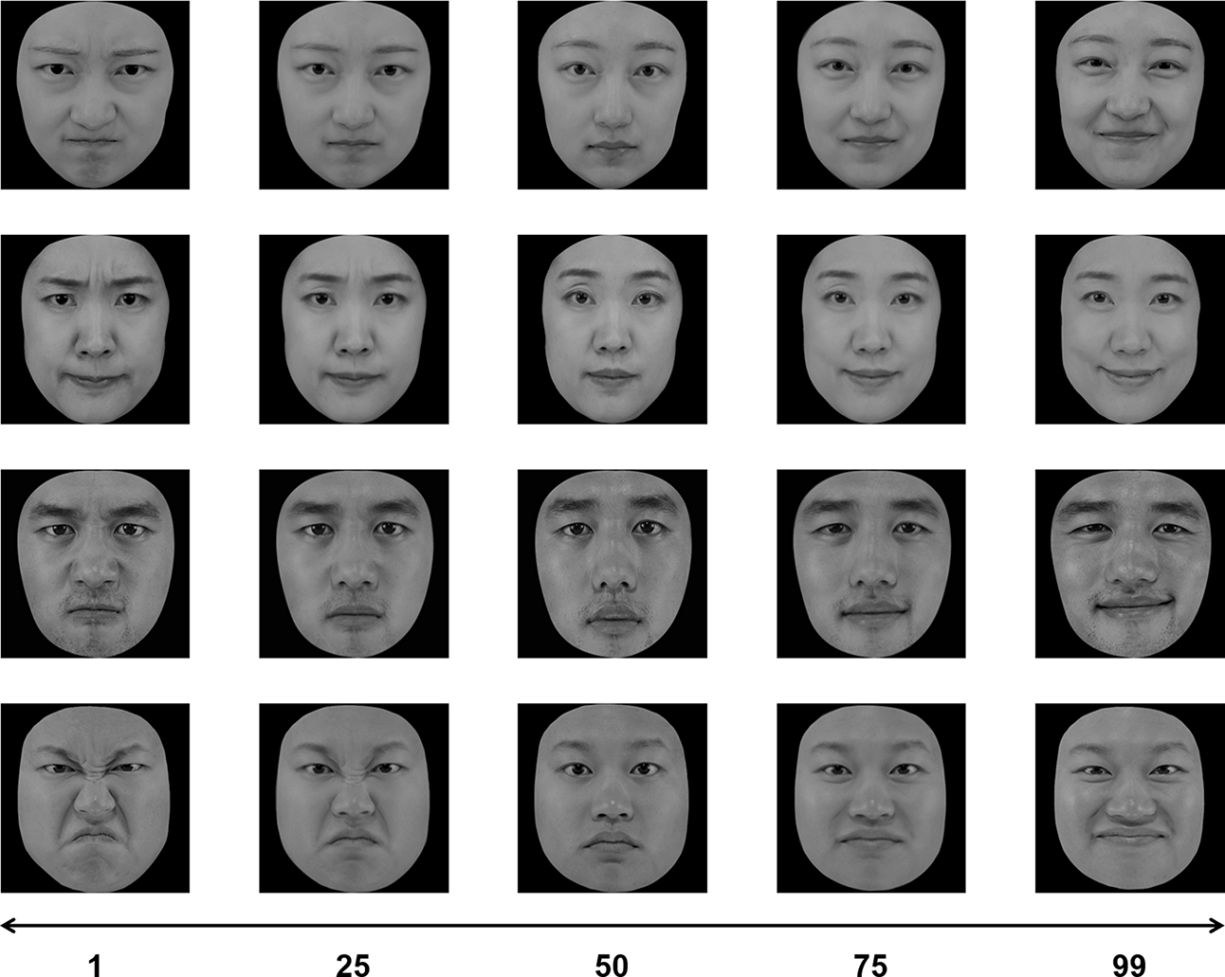
An example of face stimuli used in the study. For each of the four identities, the faces were morphed from the angriest (Face 1) to the neutral face (Face 50), and from the neutral face (Face 50) to the happiest face (Face 99)

The face images were generated by morphing using FantaMorph 5 (San Diego, CA). The morphing was carried out from the angriest face (Face 1) to the neutral face (Face 50), and from the neutral face (Face 50) to the happiest face (Face 99), for each face identity separately (Fig. 1). The difference in the emotional intensity of adjacent faces was one emotion unit (e.g., Face 1 is angrier than Face 2, and Face 99 is happier than Face 98). The greater the difference in emotional intensity between any two faces of the same identity, the easier it was to distinguish them. A previous study confirmed that emotional units were correlated with the mean emotional judgments of faces [34].

Each face set had four faces of the same identity, all showing angry or happy expressions with various intensities. The ranges of the mean emotion units were 21–30 and 70–79 for the angry and happy face sets, respectively. For each trial, the face identity and mean emotion of the set were determined at random within the range, and four unique faces were chosen around the selected mean, with 12 emotion units between two adjacent faces (i.e., mean ± 18, mean ± 6). For example, if the mean emotion was 20, then the emotional units of the four faces in the set would be 2, 14, 26, and 38. The mean emotion was never present in the set. The four faces were located randomly in an invisible 2 × 2 matrix (6.26°×7.82°) in the center of the screen.

The string was composed of six uppercase English letters, of which one contained X and was combined with K, M, N, Z, and W; the other did not contain X and was composed of H, K, M, N, Z, W. The order of the six letters in the strings was generated randomly in advance by a custom Python script, and a string was randomly selected from them in each trial. The two kinds of strings were both colored in two ways: one with all letters in red (*R* = 255, G = 0, B = 0), and another with all letters in blue (*R* = 0, G = 0, B = 255). The string stimulus was presented in the center of the screen at a visual angle of approximately 6°×1°.

### Apparatus and procedure

Participants sat at an approximate viewing distance of 60 cm in front of the monitor. The monitor was an ASUS PG279Q 27’’ IPS with a resolution of 1280 × 1024 pixels and a refresh rate of 100 Hz. The experiment was programmed in PsychoPy 2022.1.3. The main experiment lasted about 45 min, and participants had to use the mouse to complete the dual task including judgments about the strings and the facial emotions. At the beginning of each block, participants were told whether they would be asked to judge the letter in the string or the color of the string, and whether to estimate the mean emotion or the individual emotion. The importance of the string task was emphasized, which was aimed to direct attention away from the emotion task as much as possible. Only during the practice stage was feedback provided. Participants were encouraged to rely on their first impression and not to think extensively about emotional judgments [34].

**Fig. 2:**
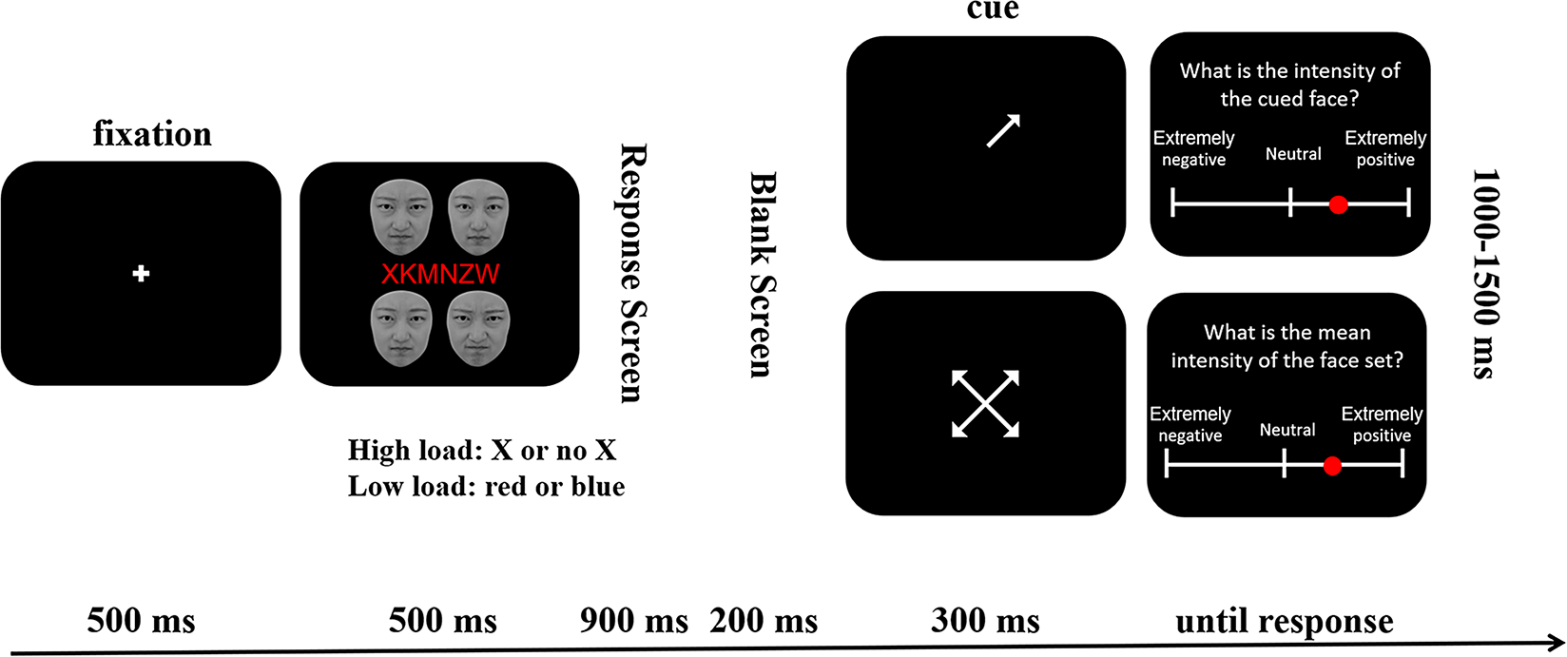
The procedure of the dual task. After fixation, a letter string and four faces were presented at the same time. Participants were required to discriminate the color of the string (low attentional load) or whether there was an X in the string (high attentional load) during the presentation of the stimuli or at the response screen. Then, a blank screen was shown, followed by a cue indicating the four faces or the single face to be judged. Next, participants estimated to (mean) emotion of the cued face(s) on the VAS. The next trial began (randomly varying between) 1000 ms– 1500 ms after participants responded

The trial began with a fixation cross at the center of the screen for 500 ms. Then the string and the face set were shown for 500 ms at the same time. The string was located at the center of the screen, with four faces surrounding it. Participants were required to discriminate the color (low attentional load) or whether there was an X in the string (high attentional load). After the stimuli vanished, a blank screen was shown for 900 ms (Fig. 2). Participants could respond to the string when the stimuli were present or during the blank screen, by pressing the left or the right button on the mouse. The corresponding correct buttons (*left* = “X present”, *right* = “X absent” in the string task; *left* = “red”, *right* = “blue” in the color task; or vice versa) were counterbalanced across participants. The maximum time window for the string judgment was thus 1400 ms. It was based on our pilot study where an independent group of 16 participants did the string task only and the response time exceeding 1400 ms was considered as an outlier (mean + 3 SD). The presentation time for the blank screen was fixed, namely, it did not disappear when participants responded. It was aimed to keep the interval the same between the presentation of face stimuli and the following emotional judgments in each condition.

After the first blank screen, another blank screen was shown for 200 ms which could provide a buffer for the cue that appeared next. The cue was presented at the center of the screen for 300 ms. In the mean emotion task, the cue consisted of four arrows pointing to the locations where the four faces appeared. In the individual emotion task, the cue was an arrow pointing randomly to one of the four locations (Fig. 2). Participants had to rate the mean emotion intensity of the four faces in the set (mean emotion task) or the emotion intensity of the cued single face (individual emotion task) by clicking the mouse on the visual analog scale (VAS). The anchors were labeled *Extremely Negative* and *Extremely Positive*, and the middle point was *Neutral*. The labels were counterbalanced across participants. The speed of response was not emphasized. The next trial started automatically 1000 ms-1500 ms after participants gave a response on the VAS. The two levels of attentional loads and two types of emotion tasks were blocked, and the order of the four blocks was counterbalanced across participants. There were 80 trials in each block. Within each block, the sets containing happy or angry faces with various intensities were also blocked. Participants were explicitly told what kinds of emotions the faces would convey at the start of each block and they needed to judge the mean/individual emotion from extremely negative to neutral for angry faces (half the scale) and from extremely positive to neutral for happy faces (half the scale). Blocking the two emotional sets was aimed at making participants focus on evaluating the emotion intensities rather than emotional valences. Before the main experiment, participants were shown the happiest, the angriest, and the neutral expression of each face identity which they could use as a reference when judging emotions on the VAS [34].

After the main experiment, we asked participants to do the mean emotion task without the concurrent string task (i.e., no attentional load), which was aimed at serving as a baseline. Participants were required to rate the mean emotion intensity of the face sets shown in the main experiment. At first, there was a fixation for 500 ms, then the face set presented at the same location in the main experiment for 500 ms. Participants rated the mean emotion intensity of the face set by clicking the mouse on the VAS after it vanished. The next trial started automatically 1000 ms-1500 ms after participants gave a response on VAS. The post-test lasted about 10 min.

### Data analysis

#### Data Conversion

The actual positions where participants clicked on the VAS in the mean emotion task or the individual emotion task were converted to data ranging from 1 to 50, and so were the emotional units of the series of morphed face stimuli. For the angry faces, the original units (1: angriest, 50: neutral) were subtracted from 51, and for the happy faces, the original units (50: neutral, 99: happiest) decreased by 49. After conversion, the more neutral the participants perceived the faces, the lower the value, and the more emotional (either positive or negative) the participants perceived, the higher the value.

We calculated the absolute difference scores as the performance index of emotion judgments. The scores were computed by subtracting the converted rating data on the VAS from the arithmetic mean emotion units of the face set in the mean task, and from the emotion unit of the cued single face in the individual task. The larger the absolute difference scores, the worse the performance of emotion judgments. To estimate the potential response biases, we also computed the signed difference scores between participants’ responses and the objective mean/individual emotion units. For both emotions, positive difference scores indicated overestimation for the emotion intensities, and negative ones indicated underestimation.

#### Data trimming

In the string task, response time exceeding ± 3 SDs of the mean RT for each participant and each condition were excluded (1%) when analyzing accuracy. No responses (1%) and incorrect responses (7%) were additionally removed when analyzing the reaction time for the correct responses. All these trials implied that attention might not be sufficiently allocated to the string task which could unexpectedly influence the concurrent emotion task and therefore were also removed when analyzing the emotion judgments. The data trimming procedure was determined and conducted before running formal data analyses.

#### Data Analysis

To verify whether the manipulation of the attentional load was effective, accuracy and RTs for the correct responses in the string task were first analyzed using a two-way repeated-measure ANOVA. The two within-subject factors were attentional load (low, high) and task type (mean emotion, individual emotion).

To examine the effect of attentional load on the performance of mean and individual emotional processing (i.e., absolute difference scores), we then conducted a repeated-measures ANOVA with attentional load (low, high) and task type (mean emotion, individual emotion) as within-subject factors, and an additional within-subject factor was emotional valence (angry, happy). To further investigate the role of attention load in ensemble coding of multiple facial expressions, the averaging performance in the baseline condition where no string task was required (i.e., no-load condition) was compared to that in the low-load and high-load conditions, with attentional load (no load, low load, high load) and emotional valence (angry, happy) as two factors in the ANOVA. A Greenhouse-Geisser correction was used when assumptions of sphericity were violated, and the uncorrected degrees of freedom were reported. Bonferroni corrections were used when multiple comparisons were performed. A similar ANOVA with attentional load, task type, and emotional valences as factors was conducted on the signed difference scores to explore the response biases.

In addition, to compare performance in the mean and individual emotion tasks to chance level, nonparametric bootstrap tests were used separately for the two emotion tasks and the two load conditions. Similar to what was done in [40], a null distribution generated by random guessing to determine the chance level was constructed by randomly shuffling the mapping between the mean of face sets (mean task) or the emotion of the cued face (individual task) and participants’ actual ratings, and then recalculating the absolute difference scores 1000 times with replacement. The *p*-value was computed as the probability of the mean error (i.e., absolute difference score) of each of the 1000 permutations being lower than that of the observed ones. If the *p*-value was below 0.05, it was assumed that the observed performance was significantly above the chance level.

Furthermore, the ability to extract mean emotion from multiple facial expressions and to process emotions individually was examined by assessing whether participants’ emotion ratings varied with the mean emotion units of each face set and with the individual emotion units of each target face using multilevel analyses for the mean task and the individual task, respectively. Random intercepts and random slopes of mean/individual emotion units for each participant were included with the nlme R package [41]. Similar as in [34], the null model with no fixed effects was first set up, and then the fixed effects of mean/individual emotion units, attentional load, and emotional valence were added to the model successively, and the interaction between mean/individual emotion units and attentional load, and between mean/individual emotion units and emotional valence were added finally. Whether the added component contributed to the mean/individual emotion ratings was tested by comparing each model to the previous model with the likelihood ratio tests. The coefficients of the final model with the smallest AIC [42] which was assumed to have the best goodness of fit were reported.

## Result

### String judgments

**Fig. 3:**
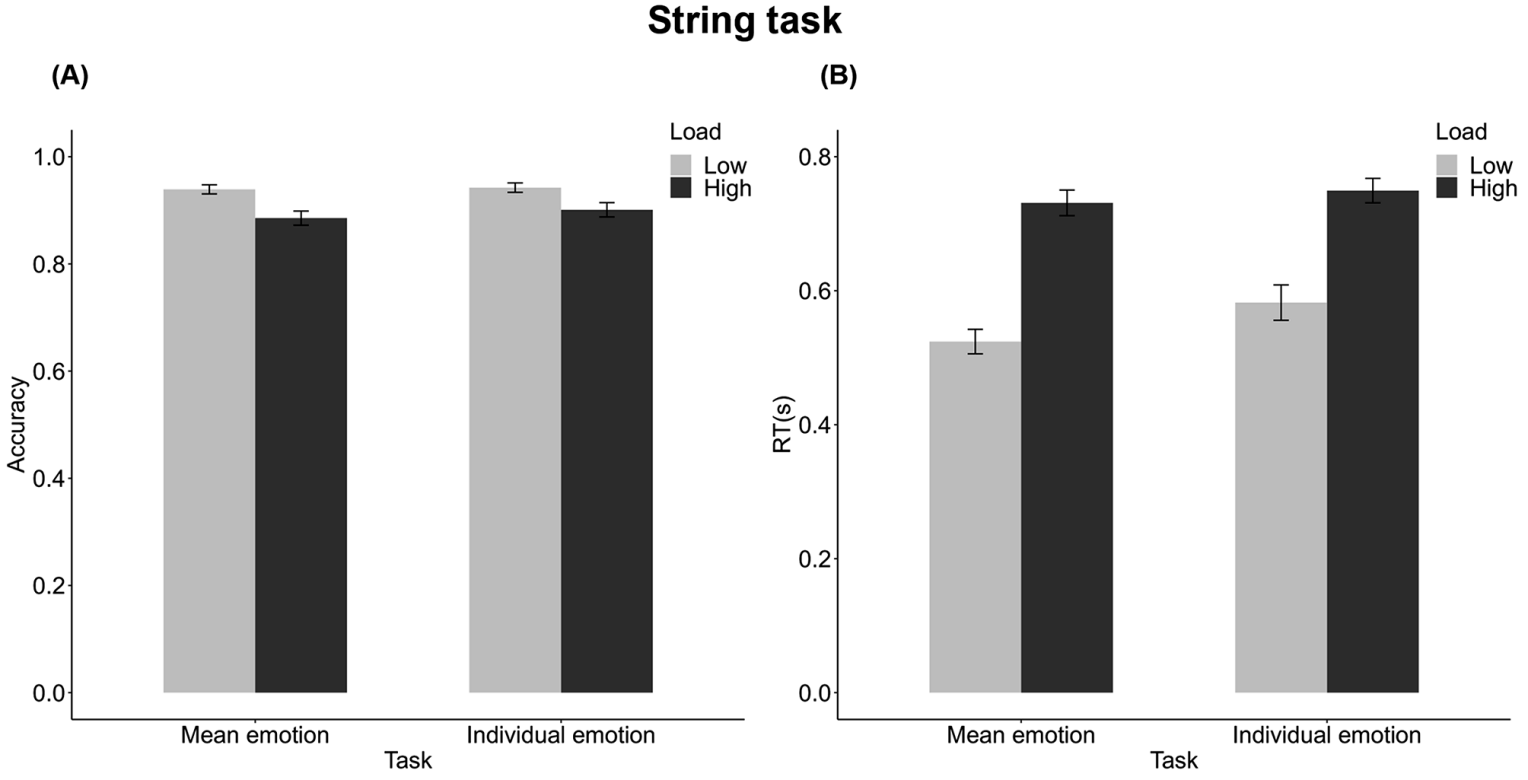
Results of (**A**) accuracy and (**B**) response time for the correct responses in the string task. The accuracy was lower and response time was slower in the high attentional load than in the low attentional load condition

Two-way repeated-measures ANOVA was used to compare accuracy in the string task under two attentional loads (low, high) and two task types (mean task, individual task). The main effect of the attentional load was significant, *F*(1, 23) = 17.73, *p* <.001, ${\eta }_{p}^{2}$= 0.44. The accuracy of string judgments was significantly higher when the attentional load was low (*M* = 0.94, *SD* = 0.04) relative to high (*M* = 0.89, *SD* = 0.05) (Fig. 3A). The main effect of the task type was not significant, *F*(1, 23) <1, *p* =.351, ${\eta }_{p}^{2}$ = 0.04, nor was the interaction between attention load and task type, *F*(1, 23) <1, *p* =.465, ${\eta }_{p}^{2}$ = 0.02.

A similar ANOVA was conducted on correct RTs of the string judgments. Results revealed that there was a significant main effect of attentional load, *F*(1, 23) = 96.85, *p* <.001, ${\eta }_{p}^{2}$= 0.81. The response time in the high-load condition (*M* = 0.74, *SD* = 0.09) was significantly slower than that in the low-load condition (*M* = 0.55, *SD* = 0.11) (Fig. 3B). There was also a main effect of task type, *F*(1, 23) = 9.73, *p* =.005, ${\eta }_{p}^{2}$= 0.30, and an interaction between attentional load and task type, *F*(1, 23) = 4.30, *p* =.049, ${\eta }_{p}^{2}$ = 0.16. Under the low attentional load, the response time of string judgments was slower when the concurrent task was the individual emotion task (*M* = 0.58, *SD* = 0.13) relative to the mean emotion task (*M* = 0.52, *SD* = 0.09); while under the high attentional load, the response time did not differ significantly between the mean (*M* = 0.73, *SD* = 0.09) and the individual tasks (*M* = 0.75, *SD* = 0.09).

### Mean and individual emotion judgments

**Fig. 4:**
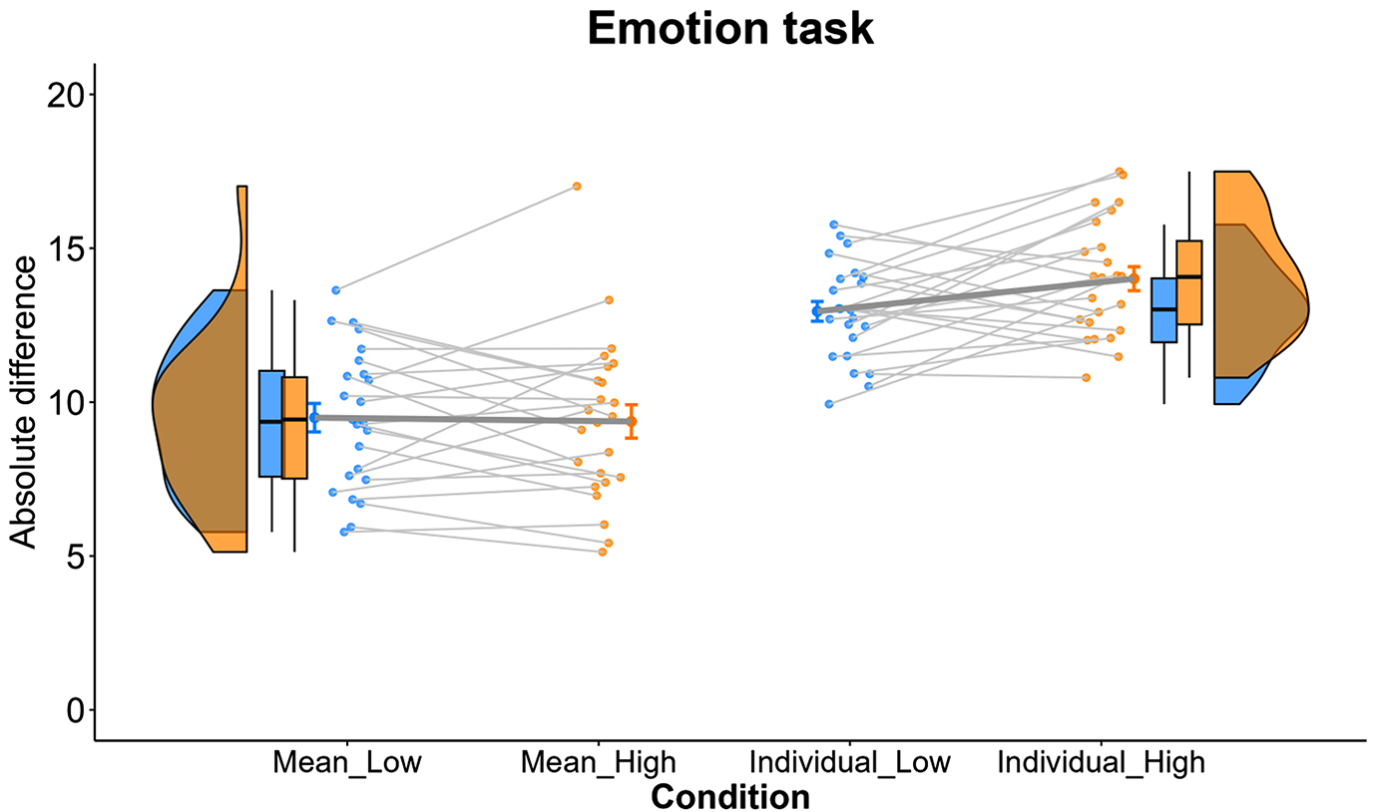
Results of the absolute difference scores in the mean emotion task and the individual emotion task under the low and high attentional loads. Each dot represents the data of each participant. Violin plots and box plots in each condition were shown beside the data points. The bold line connects the mean values in the low and the high attentional load conditions for two emotion tasks, respectively

The ANOVA on absolute difference scores revealed that there was no three-way interaction between attentional load, task type, and emotional valence, *F*(1, 23) < 1, *p* =.556, ${\eta }_{p}^{2}$ = 0.02. The main effect of task type was significant, *F*(1, 23) = 94.75, *p* <.001, ${\eta }_{p}^{2}$ = 0.81, suggesting a general advantage of extracting mean emotion from multiple faces (*M* = 9.42, *SD* = 2.33) over extracting emotion from individual faces (*M* = 13.44, *SD* = 1.45). The main effect of attentional load was not significant, *F*(1, 23) = 1.24, *p* =.276, ${\eta }_{p}^{2}$ = 0.05, but importantly there was a significant interaction between attentional load and task type, *F*(1, 23) = 6.22, *p* =.020, ${\eta }_{p}^{2}$ = 0.21. Simple main effects analysis showed that the performance of mean emotion judgments did not differ between the high-load condition (*M* = 9.37, *SD* = 2.73) and the low-load condition (*M* = 9.47, *SD* = 2.31), *p* =.744, Cohen’s *d* = 0.04. By contrast, the performance of individual emotion judgments was significantly worse under the high attentional load (*M* = 13.91, *SD* = 1.96) than under the low attentional load (*M* = 12.98, *SD* = 1.67), *p* =.049, Cohen’s *d* = 0.32. In addition, in both the high attentional load and low attentional load conditions, performances in the mean emotion task were better than those in the individual emotion task, *ps* < 0.001. Moreover, compared to the low attentional load condition, the advantage of mean emotion judgments over individual emotion judgments was larger when the attentional load was high, *p* =.023, Cohen’s *d* = 0.50 (Fig. 4).

The main effect of emotional valence was also significant, *F*(1, 23) = 19.24, *p* <.001, ${\eta }_{p}^{2}$ = 0.46. Participants’ performance was better in judging emotions from happy faces (*M* = 10.84, *SD* = 1.54) than from angry faces (*M* = 12.07, *SD* = 2.00). The interaction between emotional valence and task type was not significant, *F*(1, 23) <1, *p* =.363, ${\eta }_{p}^{2}$ = 0.04, nor was the interaction between emotional valence and attentional load, *F*(1, 23) = 1.75, *p* =.199, ${\eta }_{p}^{2}$ = 0.07.

Notably, the lack of difference in the averaging performance between the low-load and high-load conditions was not due to performance at the chance level. The permutation analysis showed that the performance of mean emotion judgments was significantly above the chance level (27.25, mean of permuted distribution) in both the low and high attentional load conditions, *ps* < 0.001. The performance of individual emotion judgments was also above the chance level (30.28, mean of permuted distribution) under both attentional loads, *ps* < 0.001.

**Fig. 5:**
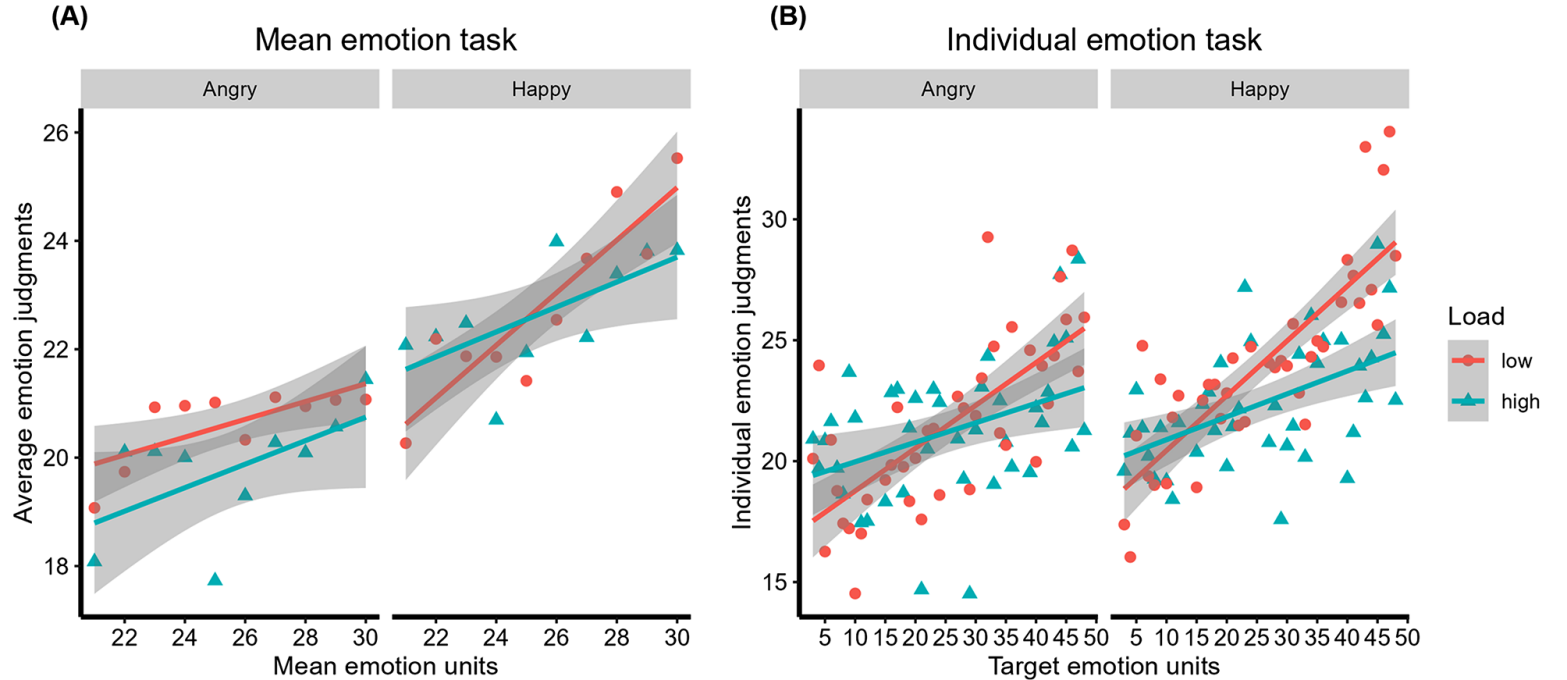
Average emotion judgments (means) in the mean emotion task (**A**) and individual emotion task (**B**), shown separately for each mean/target individual emotion unit, the two emotions and two attentional loads, collapsed across participants. The larger the judgment, the stronger emotion intensity (either happier or angrier) participants perceived the faces; the smaller the judgment, the weaker emotion (more neutral) participants judged. The regression lines in the graph were fitted for the aggregated average emotion judgments for each emotion and load condition, for illustration purposes

The multilevel analyses further confirmed that participants could indeed extract mean emotion from multiple facial expressions and to process face emotions individually. In the mean emotion task, there was a significant effect of mean emotion units, χ^2^ (6) = 19.45, *p* <.001. Adding the fixed effect of attentional load did not further improve the model, χ^2^ (7) = 1.33, *p* =.250, but adding the fixed effect of emotional valence did, χ^2^ (8) = 63.43, *p* <.001. None of the interactions contributed to the mean emotion judgments, *ps* > 0.360. Thus, the final model included only the fixed effect of mean emotion units and emotional valence. Mean emotion units positively predicted participants’ mean emotion judgments, *b* = 0.28, *SE* = 0.05, *t*(3433) = 5.20, *p* <.001. When faces in the set showed stronger mean emotions, the participants judged the mean emotion to be stronger (i.e., more positive or more negative in separate emotion blocks). In addition, happy faces (*M* = 22.71, *SE* = 0.91) were generally perceived as stronger than angry faces (*M* = 20.22, *SE* = 0.91) (Fig. 5A).

In the individual emotion task, individual emotion units of the target face also predicted participants’ emotion judgments significantly, χ^2^ (6) = 30.39, *p* <.001. There were also effects of attentional load, χ^2^ (7) = 5.39, *p* =.020, and emotional valence, χ^2^ (8) = 22.54, *p* <.001. Adding the interaction between individual emotion units and attentional load further improved the model, χ^2^ (9) = 13.75, *p* <.001, but the interaction between individual emotion units and emotional valence did not, χ^2^ (10) = 1.23, *p* =.267. When the target face expressed stronger emotion, the participants judged the face to be emotionally stronger. Interestingly, the positive relationship between individual emotion units and emotion judgments was steeper under low attentional load (*b* = 0.19, *SE* = 0.02, *t*(1756) = 8.79, *p* <.001), relative to high attentional load (*b* = 0.09, *SE* = 0.02, *t*(1696) = 4.56, *p* <.001) (Fig. 5B). The individual emotion judgments were generally stronger when the attentional load was low (*M* = 22.73, *SE* = 0.80) than high (*M* = 21.89, *SE* = 0.80). Similar to the mean emotion judgments, when the individual face showed a happy expression (*M* = 23.16, *SE* = 0.80), it was perceived as stronger than showing an angry expression (*M* = 21.46, *SE* = 0.80).

Last but not least, we compared the averaging performance among the no-load condition where no concurrent task was required, the low-attentional load, and the high-attentional load conditions. The results showed that there was no significant main effect of attentional load, *F*(1, 23) < 1, *p* =.874, ${\eta }_{p}^{2}$ < 0.01. To be more specific, the performance of evaluating the mean emotion from multiple facial expressions in the dual task did not differ from that in the single task (*M* = 9.31, *SD* = 2.35). The main effect of emotional valence was again significant, *F*(1, 23) = 15.10, *p* <.001, ${\eta }_{p}^{2}$ = 0.40, and there was no significant interaction between attentional load and emotional valence, *F*(1, 23) = 1.74, *p* =.190, ${\eta }_{p}^{2}$ = 0.70.

### Signed difference scores

The ANOVA on signed difference scores showed a significant main effect of emotional valence, *F*(1, 23) = 19.03, *p* <.001, ${\eta }_{p}^{2}$ = 0.45. The signed difference scores were smaller for the angry faces (*M* = -4.94, *SD* = 3.59) than for the happy faces (*M* = -2.23, *SD* = 3.99), and both were significantly smaller than zero, *t*(23) = -6.74, *p* <.001, Cohen’s *d* = 1.37, *t*(23) = -2.74, *p* =.012, Cohen’s *d* = 0.56. The signed difference scores were computed by subtracting the emotional units of the faces from participants’ emotional judgments, and negative ones suggested that participants tended to judge the faces to be weaker than their actual emotional units. The main effects of attentional load or task type, or any interactions between the factors were not significant, *p*s > 0.196.

## Discussion

The current study investigated whether and how attentional load impacts ensemble and individual coding of multiple facial expressions. The results showed that the performance of estimating mean emotion did not differ between the low attentional load and the high attentional load condition, suggesting that ensemble coding of multiple facial expressions requires a rather small amount of attention if any. By contrast, estimating emotion from an individual face was impaired under high attentional load relative to low attentional load, implying that individual coding and ensemble coding of facial expressions are differentially affected by the availability of attentional resources. In addition, we found that there were smaller errors (i.e., better performance) in evaluating happy over angry faces, regardless of attentional load.

### Effective manipulation of attentional load

Lavie and colleagues [26, 27] originally manipulated perceptual load by either changing the number of items for the same task or the same number of items, modifying the processing demand of the perceptual task. The latter type of manipulation is also commonly referred to as attentional load [28–30, 41], which was applied in the current study. We manipulated attentional load by asking participants to discriminate the color of the string or to detect the target letter in the string [31, 32], concurrently with an emotional judgment task. The stimuli were physically identical in the two string judgment tasks, but the task demands were different [29, 30]. Relative to the color task, the letter task was assumed to be more difficult and consume more attentional resources and thus served as the relatively higher attentional load condition. Similar to the previous studies using the dual-task design [4, 12], the accuracy of the string judgments was emphasized. We found that the accuracy of both kinds of string judgments was quite high, but accuracy was relatively lower and reaction time was longer in the letter task (high attentional load) than in the color task (low attentional load). The results confirmed an effective manipulation of attentional load. The attentional resources that could be spilled over to processing emotional expressions would be less (if any) under the high attentional load condition, compared to the low attentional load condition [26].

Previous studies have used various ways to manipulate attentional load either directly or indirectly [43], resulting in different levels of attentional resources that could be used to process the stimuli-of-interest. For example, pre-cueing certain locations or certain features of objects to be averaged provided full or most attention on them, while post-cue imposed divided attention across multiple locations or features which thus suffered from reduced attention [6, 15, 16, 44]. The dual-task design followed a similar logic where the concurrent primary task taxed the limited attention capacity and less attention could be used to do the secondary task (task-of-interest) compared to the single task without the concurrent task [45, 46]. Task relevance was also used to manipulate attention where (almost) all attention was focused on the relevant task and limited attention was available for the irrelevant task [17]. Different manipulations for attention might supply various levels of resources for the stimuli-of-interest, and may lead to different findings regarding the role of attention in ensemble coding, which we will discuss more as follows.

### Ensemble coding of facial expressions under low and high attentional load

In the current study, we found that averaging performance indicated by the absolute difference scores between participants’ estimates and the objective emotion units of the face sets [34] did not differ significantly between the low and high attentional load conditions. Importantly, the lack of load effect was not due to floor performance, since the observed performance was significantly above the chance level. Further evidence of the ability to perceive mean emotion was found in the multilevel analysis, which revealed a positive relationship between participants’ judgments and the mean emotion units of the facial expressions. Interestingly, consistent with the results of the absolute difference scores, the positive relationship was not significantly modulated by attentional loads, although some trend of difference between low and high load conditions seemed to emerge as seen in Fig. 5. Moreover, we also had a single task (no-load) as a baseline where only mean emotion was required to be extracted without the string task. To be noted, the face stimuli were immediately followed by the response screen in the single task, and the shortened interval between them relative to the dual task might make the single task much easier. Strikingly, the performance of mean emotion judgments under no load did not differ between either the low-load or the high-load conditions. The results imply that only a small amount of attention if any is required in the ensemble coding of multiple facial expressions.

The current results were consistent with our previous study and several other studies which showed that summary statistics could be extracted with minimal attention [4, 6, 12, 14]. For example, attention to the faces in the invalidly cued location was less than those in the validly cued location, yet the ability to discriminate the mean emotion from them was similar, suggesting that mean emotion could be perceived when spatial attention is limited [14]. The patients with unilateral spatial neglect whose attention could not be attracted to the stimuli in the contralateral side of their brain damage (i.e., the neglect filed) were still able to average the size of the “neglected” objects, providing another neuropathological evidence for the relative independence between focused attention and average perception [47, 48].

On the other hand, there are also studies indicating that ensemble coding requires more attention than previously thought, and substantial costs of divided attention were found in ensemble coding [15–17, 44]. Noteworthy, there are several important differences between the current study and these studies, which might contribute to the discrepant results. One is the nature of ensemble coding: a single ensemble vs. multiple ensembles. In [16], both the mean color of the circles and the mean orientation of the rectangles could be asked to judge in the post-cue condition, therefore observers had to process two ensembles simultaneously, relative to the condition where only one ensemble needed to be processed by pre-cueing one of the targets. Similarly, in [15], observers were asked to attend to both the emotion and orientation of the faces in the post-cue condition while they only needed to evaluate the mean emotion of the faces in the baseline condition. Costs of dividing attention seem to occur when computing multiple means from different groups of objects [16, 44] or different dimensions within the objects [15]. In the current study, the mean emotion task was run in blocks, and apart from the string task, participants only had to establish a single ensemble representation for the set of faces, which was found to be resistant to divided attention.

Another important difference between the current study and the previous studies which showed dependence on attention in ensemble coding was the task relevance of the ensemble task. When ensemble perception was task-irrelevant, attention was assumed to be more efficiently directed away from the ensemble-related stimuli or features [17], compared to the condition where ensemble judgments were asked throughout the task as in the current study (also see [13, 14]). For example, in an inattentional blindness study, letter recall was asked constantly while ensemble perception of the color or size of the letters was maintained task-irrelevant until the last surprise trial. The results showed that the ensemble judgment was not above chance, suggesting that some amount of attention was necessary for perceiving ensemble statistics [17].

In the current study, we did not argue that attention is entirely not required in ensemble coding of multiple facial expressions, especially considering that the levels of low and high attentional loads might not cover the full range of the attentional zoom lens [17]. Instead, based on the effective manipulation of the attentional loads as discussed above and the results that attentional loads did not modulate mean emotion processing, we assumed that a rather small amount of attention resources distributed to the expressions could result in well-above-chance averaging performance. Future studies can further explore the lower and upper bound of ensemble coding regarding their (in)dependence on attention.

### Processing of mean emotion versus individual emotion

We also examined individual emotion processing under the low and high attentional load. In the individual emotion task, four faces were present as in the mean emotion task, but participants were not asked to extract the mean emotion but needed to process the four faces individually since they would be asked to estimate the emotion of one cued single face. In contrast to the no impact of attentional load on mean emotion processing, the individual representation was worse (i.e., larger absolute difference, and lower relationship between individual emotion units and emotion judgments) in the high load compared to the low load condition, suggesting that the interference from the high attentional load was stronger for processing facial emotions individually, compared to perceptual averaging of them.

There was better performance in evaluating the mean emotion than the individual emotion, especially under the high attentional load. The range of emotional units in the individual emotion task was larger than that in the mean emotion task due to the nature of our experiment design (also see [9, 49]). With more data points covering a broader range of emotional units, the multilevel analyses might have more statistical power to detect the effect of attentional loads on the relationship between emotion units and emotion judgments in the individual task. The participants’ responses would also be naturally more dispersed when judging the emotion of the target individual faces relative to the mean emotion of the faces, which might lead to more errors in the former task. However, the reported standard error in the individual task was smaller when considering the individual differences across trials and participants in the multilevel analyses. Moreover, the advantage of mean emotion processing over individual emotion processing became even larger under the high attention load compared to the low attention load. Thus, we assume that the different impact of attentional load on the two emotion tasks could not be easily explained by the mere differences in the emotion ranges and response patterns if any. The level of difficulty of mean emotion and individual emotion processing can be better controlled in future studies to further exclude this confound.

The advantage of averaging is in line with our hypothesis and is also consistent with previous studies [1, 38]. Extracting the summary statistics from a set of multiple items can be rather precise even when representation for individual objects is impoverished by comparison. This phenomenon was found in both lower-level features, like position [4] and size [5], as well as higher-level information such as facial expression [8, 9] and face identity [50]. We previously also found that reducing attention to the faces in the periphery was more detrimental to individual emotion judgments than the mean emotion judgments [14]. Specifically, the individual emotion task contained one intact face (to be evaluated) and three scrambled faces in the target set, and both searching and emotion discrimination might be involved [14]. In the current study, all four faces might be the candidates to be judged and participants had to process all of them individually, like the member identification task [5, 9]. The faces were presented closer to the center relative to [14], but participants might have to also rely on the peripheral vision to process the faces when the central attention was focused on the string task, especially under the high attentional load. When attention is limited, individual representations are perhaps too noisy [1], or compulsorily combined into an ensemble perception [51]; on the other hand, the uncorrelated or random noise of multiple individual measurements can cancel each other out when the visual system collapses across those individual features, and thus a benefit of averaging occurs [1].

### Effect of the emotional valence on emotion perception

The effect of emotional valence was additionally explored in the current study. The faces in each set showed either happy or angry expressions with various intensities in separate blocks. We found that both happy and angry faces were underestimated relative to their objective emotion units. All face stimuli used in the study have closed mouths so that they might not be perceived as strong as the assigned unit indicated. In addition, participants perceived happy faces to be stronger and the extent of underestimation was weaker for happy faces, relative to angry faces. They also performed better in evaluating emotions from happy faces than from angry faces in both the mean and individual tasks, under both the low and high attentional load.

For the emotional processing of a single face, an attentional bias is usually found for the negative relative to the neutral or positive faces [52–54]. However, evidence also shows an advantage of recognizing a happy expression over other expressions [55]. The current study asked participants to estimate the emotional intensities of the faces, all of which in the set were the targets showing the same valence. Thus, we seemed to have no condition under which the negativity bias was usually found, such as the presence of face distractors and competition for the attentional resources between positive and negative faces [56].

Whether and how emotional valences influence mean emotion processing has also been mixed in the literature so far. Some studies found more efficient or accurate processing of angry crowds than happy crowds [34, 36], while some showed a positivity bias or advantage in ensemble coding of emotion [33, 35, 37, 57]. There were also findings showing comparable performance in averaging happy and angry expressions [34].

The mixed results might be due to some methodological differences, including the different face stimuli used in the studies which may convey various emotional intensities and contain substantial perceptual differences [58]. Different ways to measure or quantify the advantage or bias might also drive the differences. For example, asking participants to judge whether the face set was positive or negative, one found a positive bias using point of subjective equality (PSE) as a measure [37] while another found a negative bias using criteria based on signal detection theory [36]. The emotional bias in mean emotion perception could also be flexible, depending on the task framing (e.g., positive bias in approach task while a negative bias in avoidance task, [59]). It deserves more systematic investigation in future studies whether there was a differential averaging process for the threat-related compared to the positive face stimuli, and whether it would be modulated by the attentional load.

### Limitations

We combined the dual task with attentional load manipulations in the current study and explicitly required emotional judgments for either the single face or for the multiple faces, similar to the study on the working memory load on averaging line length [60]. To strengthen the effect of the concurrent string task on taxing attentional resources, we asked participants to judge the strings first and the emotions next. The interval between the face stimuli and the emotion evaluations was relatively long, as a result, although it is matched under two attentional loads and two emotion tasks. Thus, some other processes, for example, working memory might not be easily teased apart in the current study. Online neuropsychological measures (e.g., event-related potentials, [14]) or other indirect indices of perceptual averaging (e.g., interference effect, [61]) could be used in the future.

In addition, since the letter string and the set of faces were present simultaneously in the dual task, the trade-off between processing the two kinds of to-be-judged stimuli might not be easily excluded. The response time to judge the color of the string (low load) was found to be slower when the concurrent task was the individual emotion task compared to the mean emotion task. Participants might strategically delay their responses to the string while spending relatively more time processing the surrounding individual faces. Therefore, under low attentional load, the attentional resources available for the individual task were likely more than those for the ensemble task, but even so, it did not compensate for the lower performance in the former task. The response time to judge the presence of the letter (high load) did not differ significantly between the individual and ensemble tasks, suggesting that the attentional resources spilled over to the faces under high load were comparable between the two emotion tasks. Future studies should better control the strategic shifting from one task to another task in the dual-task paradigm or apply alternative ways to manipulate attentional load (e.g., [62]).

## Conclusion

In summary, we have shown that averaging across four faces in the set was not impacted by the attentional load, suggesting that a rather small amount of attention if any is required in mean emotion processing. By contrast, the increased attentional load interfered with processing facial emotions individually. Moreover, the advantage of computing the mean emotion over extracting the emotion from individual faces was larger when the attentional load increased. The current study provides a new piece of evidence supporting the advantage of averaging and showing that the mean emotion of multiple facial expressions can be extracted with limited attention, which would contribute to our understanding of ensemble coding of emotional faces.

## Data Availability

The datasets analyzed and the analyses scripts of the current study are available in Open Science Framework at https://osf.io/3aw4d/?view_only=600a83d0a55b48469323055f2a589087. The materials of the experiment are available upon contacting the corresponding author.
